# Incarcerated Inguinal Hernia Containing a Gallstone Found Decades After a Laparoscopic Cholecystectomy

**DOI:** 10.7759/cureus.44518

**Published:** 2023-09-01

**Authors:** Catlin Orschel, Lauren Gammel, Sheree A Bray, Bracken Burns

**Affiliations:** 1 Surgery, East Tennessee State University, Quillen College of Medicine, Johnson City, USA

**Keywords:** laparoscopic cholecystectomy, hernia, small bowel obstruction, gallstones, iatrogenic gallbladder

## Abstract

Iatrogenic gallbladder perforation and subsequent gallstone spillage is a common problem in laparoscopic cholecystectomy. While most commonly asymptomatic, complications due to spilled gallstones have been reported. In this case study, we report the case of a 96-year-old female with a history of laparoscopic cholecystectomy at an unknown time who presented with an incarcerated inguinal hernia and small bowel obstruction. Imaging revealed an extraluminal radiopaque foreign body located near the hernia sac. At the time of the repair, she was found to have a single gallstone located within the hernia sac, confirmed by pathology. The hernia was repaired using Lichtenstein, and her bowel obstruction was resolved postoperatively. Although gallstone spillage from iatrogenic gallbladder perforation during laparoscopic cholecystectomy is a relatively common problem, it is rarely symptomatic and may be associated with infection, abscess, and fistula formation. A rarer complication includes the formation of hernias containing gallstones, documented in fewer than 10 cases in the literature. This case demonstrates a rare consequence of leaving behind spilled gallstones following gallbladder perforation during laparoscopic cholecystectomy. It emphasizes the importance of preventing iatrogenic gallbladder perforation and retrieving any spilled gallstones during the procedure to minimize associated complications.

## Introduction

Iatrogenic gallbladder perforation and gallstone spillage during laparoscopic cholecystectomy (LC) is a well-known complication of the procedure, with reported rates ranging from 5% to 40% [1]. However, while most often associated with infection and abscess formation, hernias containing gallstones have been found in rare instances. Here, we report a case of a gallstone located within an incarcerated inguinal hernia.

## Case presentation

A 96-year-old female was brought from a nursing home with complaints of abdominal pain and vomiting. Her past medical history included hypertension, congestive heart failure, coronary atherosclerotic disease post-percutaneous coronary intervention with stent placement, hypothyroidism, and gastroesophageal reflux disease. Her surgical history was significant for LC at least 15 years prior. Her pain began two days prior, was located in the epigastric region, and was colicky in nature. She also reported pain in her left inguinal region where a known hernia was located. In addition, she reported hematemesis and anorexia secondary to nausea/vomiting. The patient denied any prior episodes of similar pain or any aggravating or alleviating factors. Her last bowel movement was two days before presentation, which was abnormal for the patient.

**Figure 1:**
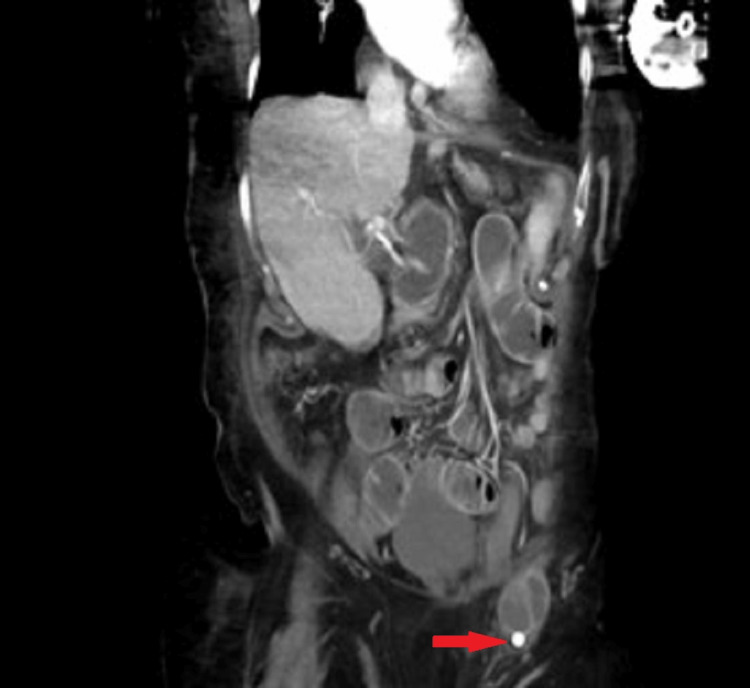
CT image shows a radiopaque shadow in the hernia sac, which proved to be a gallstone (arrow).

Her initial vital signs revealed mild hypertension, but no tachycardia or fever. She had no evidence of peritonitis or overlying skin changes. Physical examination revealed mild tenderness to palpation in the epigastric region without rebound tenderness or guarding, but severe tenderness over the left inguinal hernia that was partially reduced on the examination. Notable laboratory values included a white blood cell count of 15.1 µL and lactate of 1.3 mmol/L. A CT scan revealed a suspected small bowel obstruction with a transition point within the left inguinal hernia (Figure 1).

After initial nasogastric tube decompression, the patient was taken to the operating room for hernia repair. During the dissection, an incarcerated inguinal hernia was encountered with the small bowel and a 0.8 cm free-floating gallstone within the edge of the hernia sac. The small bowel was mildly ischemic but viable. The bowel was reduced, and the hernia sac and round ligament ligated. The hernia was repaired with a plug-and-patch mesh system.

## Discussion

Iatrogenic gallbladder perforation and gallstone spillage is a common complication of LC, occurring in up to 40% of cases [1]. Operative risk factors for iatrogenic gallbladder perforation include surgeon inexperience, the difficulty of the dissection given the presence of acute cholecystitis, palpable gallbladder preoperatively, preoperative pain for >96 hours, and right upper quadrant adhesions from prior abdominal surgeries [2]. Patient characteristics for increased risk of gallbladder perforation include advanced age, male sex, obesity, and steroid use.

Despite the frequency of gallstone spillage, most are asymptomatic. Complications due to gallstone spillage have been reported in up to 8.5% of cases where gallstones are retained [1]. Risk factors for complications due to spilled gallstones include advanced age, multiple stones, pigmented stones, stones measuring >1.5 cm, and acute cholecystitis with infected bile [3,4]. The most common complications of retained gallstones include intra-abdominal infections, abscess formation, and fistula formation [5]. There are very few literature reports documenting gallstones within hernia sacs, including inguinal hernia sacs [6-8], paraumbilical incision hernia sacs [9], and umbilical trocar site hernia sacs [10]. Gallstones can induce a strong inflammatory response leading to fibrosis and incarceration of hernias [11].

Standard of care regarding gallbladder perforation and gallstone spillage includes meticulous retrieval of all stones using sterile retrieval bags and thorough irrigation of the abdominal cavity to prevent complications. It is controversial whether or not to attempt closure of the perforation in the gallbladder before extraction. Oftentimes, given the degree of inflammation, attempted closure of the perforation is not possible and may even induce further spillage of gallstones and bile.

## Conclusions

This case highlights the frequency of gallbladder perforation and gallstone spillage during a very common procedure, the LC. It also highlights the rather rare nature of hernia sacs containing spilled gallstones. Although most often asymptomatic, spilled gallstones can produce significant morbidity to the patients even decades after the surgery. A thorough effort should be made to retrieve spilled gallstones to avoid both infectious and long-term complications for the patient.
